# Reported Hours of Sleep, Diabetes Prevalence and Glucose Control in Jamaican Adults: Analysis from the Jamaica Lifestyle Survey 2007-2008

**DOI:** 10.1155/2011/716214

**Published:** 2011-11-17

**Authors:** Chisa G. Cumberbatch, Novie O. Younger, Trevor S. Ferguson, Shelly R. McFarlane, Damian K. Francis, Rainford J. Wilks, Marshall K. Tulloch-Reid

**Affiliations:** Epidemiology Research Unit, Tropical Medicine Research Institute, The University of the West Indies, Kingston 7, Mona, Jamaica

## Abstract

*Background*. There are limited data on sleep duration and diabetes from developing countries. We therefore examined the relationship between reported hours of sleep, diabetes prevalence and glucose control in Jamaican adults. *Methods*. Data on reported hours of sleep and diabetes (based on glucose measurement and medication use) from a national survey of 15–74-year-old Jamaicans were analyzed. *Results*. The 2,432 participants (31% M, Age 42 ± 16 years, BMI 27.6 ± 6.6 kg/m^2^, diabetes prevalence 12%) reported sleeping 8.2 ± 1.8 hours. In men, sleeping less than 6 hours (OR (95% CI) = 2.65 (1.09–6.48)) or more than 10 hours (OR (95% CI) = 4.36 (1.56–12.19)) was associated with diabetes when adjusted for age, BMI, and family history of diabetes. In women sleeping less than 6 hours was associated with a reduced likelihood of diabetes after adjusting for the same confounders ((OR (95% CI) = 0.43 (0.23–0.78)). There was no significant association between sleep and glucose control. *Conclusion*. Insufficient and excessive sleep was associated with increased diabetes prevalence in Jamaican men but not women.

## 1. Introduction

Jamaica, like many other Caribbean countries, has experienced an increase in the prevalence of diabetes over the last 50 years [1]. In cross-sectional studies of selected adult populations, the prevalence of diabetes has increased—from 1% in 1960 to between 13%–17.9% in the mid-1990s [1–4]. Data from the 2008 island-wide Jamaican Health and Lifestyle survey estimated the prevalence of diabetes among 15–74-year-old Jamaicans to be 7.9% after adjusting for sampling methodology [5]. 

The role of the well-established risk factors for diabetes in Jamaica such as obesity, hypertension and family history of the disease has been investigated [4]. In the Spanish Town Study, obesity was estimated to have an attributable risk percentage of 66% for diabetes [4]. The importance of obesity has also been confirmed in prospective analysis of this data [6]. Few studies in the Caribbean and other regions undergoing the epidemiological transition have examined the contribution of novel risk factors on diabetes risk using population based data. Sleep is one such risk factor for diabetes that has not been previously explored [7]. 

In Western societies, a decreasing trend in sleep hours in the society coincides with the increasing diabetes prevalence [8]. Sleep duration extended beyond or curtailed before the normal sleep period of 7-8 hours has been associated with metabolic and endocrine changes [9, 10]. Curtailed sleep has been shown to increase insulin resistance, a precursor of type 2 diabetes [7]. Shortened sleep duration has also been demonstrated to produce an adverse hormonal profile (increase in cortisol, ghrelin, and a reduction in leptin) and increased inflammatory markers [10–12]. Sleep deprivation would therefore be expected to affect both diabetes prevalence and glycemic control. 

While several cross sectional and cohort studies have investigated the relationship between sleep duration, diabetes prevalence and glucose control, none of these studies have been conducted in black populations undergoing the epidemiological transition [13–16]. Additionally, the relationship between sleep and diabetes has not been consistently demonstrated in black populations [17]. We therefore examined the relationship between sleep duration and diabetes prevalence and glycaemic control (assessed by glycosylated haemoglobin) in Jamaican adults. We also evaluated whether any relationship between sleep and diabetes prevalence was independent of traditional risk factors for the disease such as age, obesity and family history of diabetes.

## 2. Methods

The Jamaican Health and Lifestyle Survey II was a cross-sectional survey conducted by the Epidemiology Research Unit of the Tropical Medical Research Institute (TMRI) and the Ministry of Health, between 2007 and 2008 [5]. The study sample was selected using a multistage cluster sampling methodology. The primary sampling units were enumeration districts randomly selected from each of the fourteen parishes in the island using probability proportionate to size. Within each enumeration district, systematic random sampling was used to select households beginning at a random starting site. Within each household, a single respondent between the ages of 15–74 was selected using the Kish methodology [18]. Once an eligible individual was selected to participate in the study, interviewers were required to revisit households where the participant was not home at the time of first contact three times before the household participant was deemed a refusal. Approximately 30 participants per enumeration district were selected resulting in a final sample of 2848 persons. The refusal rate was less than 2%. 

The survey included an interviewer administered questionnaire where information on demographics, education, socioeconomic status, personal and family medical history and emotional health was collected. Participants were asked about their usual bed times and times of waking up. They were also asked whether they woke up several times during the nights. Trained interviewers measured height in centimetres using a portable stadiometer and weight in kilograms using an electronic digital scale. The body mass index (BMI) was calculated using the weight (kilograms) divided by height (metres) squared. Blood pressure was measured with a mercury sphygmomanometer following a standardized protocol [19]. Participants were seated for 5 minutes before measurement and 3 measurements (each one minute apart) were performed. The average of the last two readings was used in analyses for blood pressure.

Participants were visited on a subsequent day in order to obtain fasting finger-stick blood samples for glucose using an Accutrend Glucometer (Roche Diagnostics GmBh, Germany). Glycosylated haemoglobin was also measured from the capillary blood sample using a NycoCard HbA1_c_ reader (Axis Shield) in those with diabetes or an elevated fasting glucose at evaluation. 

The study was approved by the University of the West Indies, Faculty of Medical Sciences, Ethics Committee and written informed consent was obtained from each participant before enrolment. 

### 2.1. Definitions 

#### 2.1.1. Sleep Duration

Sleep duration was calculated in hours using responses to the questions “What time do you usually go to sleep?” and “What time do you usually wake up?” Sleep quality was assessed by asking the question “Do you wake up several times during your sleep?”

#### 2.1.2. Diabetes

Participants were classified as having diabetes if they had a fasting capillary glucose ≥6.1 mmol/L [20] when tested or if they responded yes to the question, “Have you been prescribed medication for your diabetes?”

#### 2.1.3. Controlled Diabetes

Participants with a glycosylated haemoglobin of 7% or less were classified as having controlled diabetes, while those with a value above 7% were classified as having uncontrolled diabetes.

#### 2.1.4. Hypertension

Participants were classified as hypertensive if their mean systolic blood pressure (SBP) ≥140 mmHg and or mean diastolic blood pressure (DBP) ≥90 mmHg or if they answered yes to the question “Have you been prescribed medication for your high blood pressure?”

#### 2.1.5. Depression

Participants were classified as depressed if they had seriously considered suicide in the last year or if they answered yes to either of the following questions “During the past month have you been bothered a lot by little interest or pleasure in doing things?” or “During the past month have you been bothered a lot by feeling down, depressed or hopeless?”. They were also classified as depressed if they answered positively to at least three of the following questions: “During the past month have you been bothered a lot by: (i) feeling sad or lonely, (ii) feeling guilty or worthless, (iii) change in appetite or (iv) change in sleeping patterns?” [21].

#### 2.1.6. Physical Activity

Respondents were categorised into three groups: high activity, medium activity or low activity/inactive-based on their response to a study questionnaire which included questions on work-related and leisure time physical activity.

#### 2.1.7. Drinker

Participants were categorised nondrinkers if they answered “no” to the question “Do you ever drink alcohol?” or if they reported abstaining from alcoholic beverages for more than a year. Participants reporting alcohol use in the last year were classified as drinkers.

#### 2.1.8. Smoker

Participants were classified as smokers if they reported smoking 100 or more cigarettes in their life. All other persons were classified as nonsmokers.

## 3. Statistical Analysis

Data were analysed using Stata Version 9.1 (Stata Corporation, College Station, Tex). Participants were divided into six groups based on the total hours spent sleeping: <6 hrs 6-7 hrs, 7-8 hrs, 8-9 hrs, 9-10 hrs and >10 hrs. Analysis was done separately for men and women as there was evidence of interaction between sleep duration and sex on preliminary analysis. 

Sex differences in participant characteristics were assessed using the Wilcoxon rank-sum test. Trends across sleep duration categories were assessed using a non-parametric test for trend. Those sleeping 8-9 hours were used as the referent group based on a smoothed plot of diabetes prevalence and hours of sleep which did not show any significant difference in the odds of diabetes in men or women sleeping between 6 and 9 hours. Logistic regression was used to assess the effect of sleep on diabetes prevalence. Adjusted odds ratios were obtained from a base model that included age, obesity and family history of diabetes. Additional confounders were added to this model and only those variables that significantly improved the model were retained. 

To assess the effect of sleep on the control of diabetes, analysis was limited to those participants who were identified as having diabetes for more than one year. Multivariable logistic regression adjusting for age, sex and insulin use, using good versus poor control as the outcome, was used to assess the effect of sleep on diabetes control.

## 4. Results

Analysis for this study was restricted to respondents who had complete data for diabetes diagnosis, sleep duration, and potential confounders. Of the 2,848 individuals who participated, 2,432 participants had complete information on the variables of interest. The final sample had a higher proportion of urban dwellers than the total sample (63% versus 57%; *P* = 0.02) but there were no other differences in the demographic factors assessed.

The population was predominantly black (94.3%), female (69%), with a mean (±SE) age of 42 ± 16 years, BMI of 27.6 ± 6.6 kg/m^2^, and reported sleep duration of 8.2 ± 1.8 hours. Approximately 12% (men 11%, women 13%) had diabetes, with 29% diagnosed with diabetes at the time of the survey. The median duration of diabetes was 7.0 years, with 18% reporting treatment with insulin. 

Table 1 presents characteristics of the population by sex. Women were more likely to have depression/depressive symptoms, live in crowded conditions, be classified as physically inactive and report a family history of diabetes. Women had a higher mean BMI and were more likely to report receiving advanced education than men. Men were more likely to smoke and consume alcohol. 

Sex specific characteristics of the sample by sleep duration category are presented in Tables 2 and 3. In both sexes, there was an inverse relationship between BMI and reported hours of sleep. In men, the proportion of alcohol drinkers was inversely associated with the number of hours of sleep. In women, age, post-secondary education and high physical activity were inversely associated with the number of hours of sleep.

In univariate analysis, using participants sleeping between 8-9 hours per night as the referent, men who reported sleeping more than 10 hours per night had an increased likelihood of diabetes (OR [95% CI] = 3.97 [1.57–10.1]). When adjusted for age, BMI and family history of diabetes the odds of diabetes increased for each of the sleep categories and also became significant in those who reported sleeping less than 6 hours with an OR [95% CI] = 2.65 [1.09–6.48] (see Table 4). Adjustment for depression, alcohol use, smoking, physical activity and education did not significantly affect these findings. 

In univariate analysis in women, there was no difference in the likelihood of diabetes in any sleep category compared to the referent group. After adjusting for age, BMI, and family history of diabetes the odds of diabetes were significantly lower in those who reported sleeping less than 6 hours (OR [95% CI] = 0.43 [0.23–0.78]) (see Table 4). The results were unchanged after adjusting this model for depression, physical activity, alcohol use, smoking, crowding and educational status. 

The relationship between reported hours of sleep and the prevalence of diabetes did not change when analysis was restricted to those participants with established diabetes or those diagnosed as having diabetes at the time of the survey, though many of the associations were no longer statistically significant due to the reduced sample size. Obesity did not modify the association between sleep and diabetes prevalence in this study. 

Reported duration of sleep and diabetes control was examined in 228 participants (26% men) who had diabetes for more than 1 year. Approximately two-thirds (69%) had an  HbA_ 1c_ less than 7.0%. Participants who had controlled diabetes were older (Median Age (interquartile range) 59 [48–68] versus 54 (44–63) years) but there were no differences in the reported hours of sleep between the groups. Using those sleeping 8-9 hours as the referent, there was no significant association between good diabetes control and sleep duration on univariate analysis. This remained unchanged after adjusting for insulin use and age (data not shown). 

## 5. Discussion

We found a significant relationship between sleep duration and diabetes prevalence among Jamaican adults, although the relationship differed between the sexes. In men, both shorter and longer hours of sleep were associated with an increased likelihood of diabetes, and this relationship remained significant after adjustment for potential confounders. Conversely in women, shorter hours of sleep were associated with a reduced likelihood of diabetes, even after adjustment for the same confounders. No association was found between sleep duration and diabetes control.

Our findings in men are consistent with those of other studies which have examined the relationship between sleep duration and diabetes. In a cross sectional study of 323 men and 417 women aged 21–64 years, Chaput found an increased likelihood of diabetes for those sleeping 5-6 hours and 9-10 hours in both sexes [22]. Gangwisch also demonstrated a similar association in his study of US National Data [23]. Other studies, such as the lifestyle survey of Korean men have also found an association between either short or long sleep duration and diabetes prevalence in men [15]. 

Contrary to what was expected, women who slept less than 6 hours had a reduced likelihood of diabetes. While some studies have found no association between sleep deprivation and diabetes [17] or sex differences in the association between short sleep duration and diabetes [13, 24], none of these studies have suggested that there might be a protective effect from lack of sleep. While these women had a higher BMI, they were more physically active and better educated. The association between sleep and diabetes was still significant after adjustment for these potential confounders. In a study of Korean men, sleep deprivation was associated with diabetes prevalence in the nonobese participants [15]; however, we found no difference in the association according to body mass index in the Jamaican women. Sex differences in the relationship between sleep and diabetes prevalence could be attributed to biological differences in sexual hormones that affect sleep or differences in stress and the stress response. This finding will require additional exploration.

Short and long sleep duration has been shown to affect metabolic and endocrine control [7, 9]. Sleep deprivation affects serum leptin (an appetite suppressive hormone) and Ghrelin (an appetite stimulatory hormone). There are few data examining the effect of sleep on these hormone levels but hours of sleep have been negatively correlated with Ghrelin levels and sleep deprivation has been shown to result in lower levels of leptin [25]. This may result in poor food choices and excessive eating. Sleep deprivation may also reduce the amount of rapid eye movement sleep, interfering with brain glucose utilization and resulting in increased serum glucose levels. Sleep disorders are associated with increased sympathetic activity, which leads to an increase in gluconeogenesis and glycogen breakdown, thus inducing insulin resistance. Additionally, sleep deprivation may also affect resting energy expenditure [25]. 

The association between sleep and diabetes prevalence may be a result of confounding from obesity. Lack of sleep as well as longer hours of sleep has also been associated with increased obesity. Obesity, a strong diabetes risk factor, can also result in poor sleep quality and interrupted sleep [10]. In our analysis, the effect of the association was still present even when adjusting for differences in BMI. Sleep apnea, which can result in insulin resistance, can also be considered another risk factor for diabetes and may result in excessive sleepiness and longer reported hours of sleep. 

Reverse causation may also result in this association. Diabetes complications are often the result of poorly-controlled long-standing diabetes. Some complications, such as peripheral neuropathy, which are worse at night, can interfere with the ability to sleep and thereby result in an association between sleep and diabetes control. Additionally, insulin, typically reserved for patients with poor glucose control, may be associated with a higher risk of hypoglycaemia, especially at nights and this may also interfere with sleeping patterns in patients concerned about this risk. The relationship between diabetes and sleep, however, was still present even when restricted only to those diagnosed with diabetes at the time of the survey suggesting that the association may not be a result of having diabetes or its treatment.

We did not demonstrate an association between sleep and diabetes control in our analysis even with adjustment for age or insulin use This may be a result of insufficient power to detect this effect. Overall glucose control was good in this sample with over two-thirds having an HbA_1c_ of 7% or less. There was no evidence that sex modified the relationship between glucose control and hours of sleep. 

While there was no statistically significant difference in the prevalence of depression or poor quality sleep with reported hours of sleep in the study, these conditions appeared to be more common in those who reported sleeping the recommended number of hours. Adjusting for depression did not make any difference in the association between diabetes prevalence and hours of sleep in the analysis. Almost half the participants reported having poor sleep quality. It was not found to be a significant predictor of the presence of diabetes, and adjusting for sleep quality did not affect the association between diabetes and hours of sleep in multivariable analysis. 

This study has some limitations. Despite attempts to obtain a nationally representative sample, the participants were predominantly female and this may suggest a frame error. The female predominance has been seen in our previous national survey [26] and may reflect the household structure in Jamaica; as for participants to be eligible for selection, they needed to spend at least 3 nights at home. In other analyses, we have corrected this by applying survey sampling weights; however, as we were interested in the relationship between sleep and diabetes, we did not make the adjustment for this paper. 

The cross-sectional design restricts the ability to infer causality as the temporal relationship between sleep duration and diabetes cannot be established; however, previously conducted cohorts have shown that sleep duration is a likely risk factor for diabetes mellitus [27]. Another limitation of the study was the inability to account for sleep apnoea and diabetic complications including chronic pain which were not measured and could potentially produce a relationship between sleep and diabetes prevalence by interfering with sleep or through changes in physical activity. The definition of diabetes was based on capillary glucose and not plasma; however, this technique still provides a useful estimate of diabetes prevalence from a large nationally representative sample and had been considered as an acceptable means for diagnosing diabetes in previous WHO criteria [20]. Glycosylated haemoglobin was measured using a point of care testing machine (NycoCard) which may tend to underestimate the true value but allows subjects to be appropriately classified for clinical purposes and provides a better estimate of overall glucose control than a single finger stick glucose measurement. Sleep duration and quality were self-reported which may be subjective, but this is considered equally effective as actigraphy in measuring sleep patterns of individuals [28]. As the assessment took place at one point in time, we may have failed to identify changes in the pattern of sleep that may be of importance in this association. 

Our findings support the growing evidence that sleep may play a role in the prevention of diabetes, particularly in black men living in developing countries. We are unaware of any other studies conducted in predominantly black developing countries to examine this issue. Adequate sleep may be an important public health initiative to stem the diabetes epidemic in the region. Further study of the reasons for the sex differences in the association between sleep and diabetes and to determine the physiological and biological mechanisms underlying the associations between sleep duration and diabetes needs to be conducted.

## Figures and Tables

**Table 1:** Characteristics of the study population by sex.

Characteristics	Men *n* = 754	Women *n* = 1678	*P* value
Age (yrs)	42.2 ± 17.0	42.8 ± 15.5	0.80^ b^
Urban (%)	57.2	57.5	0.89^ c^
Depression (%)^a^	0.8	3.9	<0.01^c^
BMI (kg/m^2^)^a^	24.7 ± 5.4	28.9 ± 6.8	<0.01^b^
Mean hours of sleep^ a^	7.8 ± 1.8	8.3 ± 1.8	<0.01
Poor quality of sleep (%)^ a^	34.8	46.4	<0.01^c^
All Diabetes (%)	10.6	13.1	0.09^c^
Newly diagnosed (%)^ a^	4.4	3.3	
Established diabetes (%)^ a^	6.2	9.8	
Diabetes Duration (yrs)	10.3	6.2	0.15^c^
Hypertension (%)	31.0	33.1	0.32^c^
Family history of diabetes (%)^a^	30.2	41.7	<0.01^c^
Highest level of education (%)^a^			0.03^c^
Primary/junior high	43.2	37.5	
Secondary	40.6	45.0	
Post-secondary	16.2	17.5	
Persons/habitable room^ a^	1.0	1.3	<0.01^b, d^
Alcohol use (%)^a^			<0.01^c^
Nondrinker	19.9	54.8	
Drinker	80.1	45.3	
Smoker (%)^a^	67.7	51.0	<0.01^c^
Physical activity (%)^ a^			<0.01^c^
High activity	47.2	18.8	
Medium activity	22.9	19.2	
Low activity/inactive	29.9	62.0	

Data are given as mean ± SD unless otherwise specified. ^a^indicates there is a significant difference (*P* < 0.05) between the sexes, ^b^Wilcoxon rank sum test, ^c^Chi square test, ^d^Median value presented.

**Table 2:** Demographic, anthropometric, lifestyle factor and chronic disease prevalence, with self-reported hours of sleep in Jamaican men.

Characteristics	Hours of sleep						*P* value
	<6 *n* = 149	6-7 *n* = 146	7-8 *n* = 177	8-9 *n* = 141	9-10 *n* = 87	>10 *n* = 56	
Age (yrs)	39	45	38	42	43	35	0.91
BMI kg/m^2^ ^a^	24.5	24.3	23.7	23.2	23.1	22.6	0.01
Depression (%)	0.0	0.7	0.6	2.8	0.0	0.0	0.16
Poor quality of sleep (%)	29.5	31.5	34.5	39.7	42.5	33.9	0.40
Diabetes (%)							
All	10.0	10.3	7.4	4.9	12.6	19.6	0.06
Established	6.0	8.9	5.7	2.8	8.0	10.7	
Newly diagnosed	4.0	1.4	1.7	2.1	4.6	8.9	
Hypertension (%)^a^	6.0	8.9	5.7	2.1	8.1	10.7	0.05
Family history of diabetes (%)	2.8	9.2	10.3	10.1	10.8	8.9	0.82
Highest level of education (%)							
Primary/junior high	34.7	47.6	39.8	44.3	50.6	50.0	0.12
Secondary	42.9	33.8	44.9	42.1	36.5	41.1	
Post-secondary	22.5	18.6	15.3	13.6	12.9	8.9	
Alcohol use (%)^a^							
Nondrinkers	10.7	20.6	20.9	24.1	20.7	30.4	0.02
Drinkers	89.3	79.5	79.1	75.9	79.3	69.6	
Smoker (%)	67.1	63.9	68.3	71.1	71.1	65.4	0.97
Physical activity (%)							
High	46.3	49.3	55.4	44.0	36.8	41.1	0.09
Medium	20.1	24.0	19.8	28.4	27.6	17.9	
Low/Inactive	33.6	26.7	24.9	27.7	35.6	41.1	

Median values presented unless otherwise stated. ^a^indicates there is a significant difference (*P* < 0.05) between the self-reported sleep categories using non-parametric test for trend of continuous variables or chi square statistic for categorical variables.

**Table 3:** Demographic, anthropometric, lifestyle factor and chronic disease prevalence, with self-reported hours of sleep in Jamaican women.

Characteristics	Hours of sleep						*P* value
	<6 *n* = 149	6-7 *n* = 146	7-8 *n* = 177	8-9 *n* = 141	9-10 *n* = 87	>10 *n* = 56	
Age (yrs)^a^	44	42	43	39	43	34	<0.01
BMI kg/m^2^ ^a^	29.1	28.9	28.5	28.4	27.5	27.4	<0.01
Depression (%)	4.3	4.3	3.0	4.9	4.5	2.0	0.51
Poor quality of sleep (%)	46.3	43.7	45.3	44.6	50.0	52.2	0.26
Diabetes (%)							
All	9.8	11.9	11.8	14.3	17.0	11.5	0.46
Established	7.0	10.0	9.7	11.1	14.4	9.5	
Newly diagnosed	2.8	1.9	2.1	3.2	2.6	2.0	
Hypertension (%)	35.1	31.0	33.2	34.0	37.0	28.9	0.49
Family history of diabetes (%)	49.3	41.3	42.1	42.8	38.4	37.6	0.82
Highest level of education (%)^a^							
Primary/junior high	35.5	33.2	36.7	38.5	42.8	38.3	<0.01
Secondary	40.2	41.8	44.7	46.6	45.7	50.3	
Post- secondary	24.3	25.0	18.6	14.9	11.5	11.4	
Alcohol use (%)							
Nondrinkers	51.4	54.0	54.7	56.5	56.1	54.0	0.89
Drinkers	48.6	46.0	45.0	43.5	43.9	46.0	
Smoker (%)	60.9	53.9	51.5	44.2	46.9	48.3	0.80
Physical activity (%)^a^							
High	21.5	24.6	21.2	18.6	12.0	14.4	<0.01
Medium	17.9	23.9	21.5	16.7	16.9	18.4	
Low/Inactive	61.1	51.5	57.4	64.7	71.2	67.2	

Median values presented unless otherwise stated. ^a^indicates there is a significant difference (*P* < 0.05) between the self-reported sleep categories using non-parametric test for trend of continuous variables or chi square statistic for categorical variables.

**Table 4:** Odds ratios (95%CI) for the effect of reported hours of sleep on risk of diabetes by sex in Jamaican adults. All models adjusted for age, BMI, and family history of diabetes.

	Reported hours of sleep					
	<6 hrs	6-7 hrs	7-8 hrs	8-9 hrs	9-10 hrs	>10 hrs
Men						
All diabetes	2.65 (1.09–6.48)	1.33 (0.52–3.42)	1.64 (0.65–4.13)	1	2.41 (0.91–6.39)	4.36 (1.56–12.19)
Established diabetes^a^	4.25 (1.01–17.92)	4.36 (1.08–17.59)	4.70 (1.14–19.35)	1	5.09 (1.15–22.62)	6.35 (1.32–30.59)
Newly diagnosed^b^	1.83 (0.63–5.31)	0.16 (0.63–5.31)	0.60 (0.16–2.20)	1	1.40 (0.41–4.86)	3.15 (0.93–10.61)
Women						
All diabetes	0.43 (0.23–0.78)	0.75 (0.45–1.26)	0.72 (0.45–1.16)	1	1.38 (0.86–2.23)	0.78 (0.43–1.41)
Established diabetes^a^	0.39 (0.19–0.81)	0.90 (0.50–1.62)	0.78 (0.45–1.37)	1	1.59 (0.91–2.77)	0.92 (0.47–1.80)
Newly diagnosed^b^	0.50 (0.19–1.32)	0.44 (0.16–1.16)	0.56 (0.25–1.27)	1	0.98 (0.43–2.21)	0.45 (0.14–1.42)

^a^Established diabetes: aware of diagnosis of diabetes prior to survey, ^b^Newly diagnosed: diabetes first recognised at the time of survey.
